# KRT6A Is a Biomarker of PAS Progression and Enhances the Invasive Ability of Trophoblast Cells

**DOI:** 10.3390/cells15131204

**Published:** 2026-07-02

**Authors:** Zhirong Guo, Jiaqi Huang, Weiran Zheng, Ruochong Dou, Xinrui Yang, Huixia Yang, Jingmei Ma

**Affiliations:** 1 Department of Obstetrics and Gynecology, Peking University First Hospital, Beijing 100034, China; guozhirong@bjmu.edu.cn (Z.G.);; 2 Beijing Key Laboratory of Innovations and Transformations in Intelligent and Precise Diagnosis and Treatment Technologies for Reproductive Health, Beijing 100191, China; 3 Department of Radiation Therapy, Peking University People’s Hospital, Beijing 100044, China

**Keywords:** placenta accreta spectrum, KRT6A, proteomics, biomarker

## Abstract

**Highlights:**

**What are the main findings?**

- Using longitudinal plasma samples from PAS patients and a DIA-based quantitative proteomic strategy, we identified KRT6A as a circulating biomarker whose expression increases significantly with PAS progression. Functional gain-of-function experiments further demonstrated that KRT6A overexpression directly enhances the invasive capacity of trophoblast cells.
- Mechanistically, bioinformatics and correlation analyses suggest that the pro-invasive role of KRT6A is mediated through the activation of the MYC signaling pathway.

**What are the implications of the main finding?**

- Our findings identify KRT6A as a circulating plasma biomarker for monitoring PAS progression, offering a potential adjunctive tool to current ultrasound-based approaches for risk assessment.
- The demonstration that KRT6A directly enhances trophoblast invasion suggests that targeting KRT6A or its downstream MYC signaling pathway may represent a novel therapeutic strategy for preventing deep myometrial invasion in PAS patients.

**Abstract:**

Placenta Accreta Spectrum (PAS) is a severe obstetric disorder characterized by excessive trophoblast invasion into the uterine myometrium, leading to life-threatening hemorrhage at delivery. While closely monitored, the molecular drivers of its progression remain poorly defined, hindering predictive and therapeutic strategies. Here, we employed longitudinal quantitative proteomics on maternal plasma from five PAS patients across gestation. We identified Keratin 6A (KRT6A) as a key biomarker whose plasma levels increase with PAS progression. Immunohistochemistry confirmed the specific upregulation of KRT6A in trophoblasts at the maternal–fetal interface in PAS. Functional studies demonstrated that KRT6A overexpression significantly enhances the migration and invasion capabilities of trophoblast cell lines in vitro, without affecting proliferation or apoptosis. Integrative bioinformatics analysis linked KRT6A to the activation of the MYC signaling pathway, a known driver of invasiveness. Our data establish KRT6A as a plasma biomarker correlating with PAS severity and a functional regulator of trophoblast invasiveness. These findings suggest a novel mechanism wherein KRT6A-mediated enhancement of trophoblast invasion, potentially via MYC signaling, contributes to PAS pathogenesis. This work provides a potential circulating biomarker for monitoring PAS progression and implicates KRT6A as a candidate therapeutic target for mitigating excessive placental invasion.

## 1. Introduction

Placenta Accreta Spectrum (PAS) refers to a group of disorders characterized by abnormal placental adhesion to the uterine wall [1]. Invasive PAS represents a significant cause of uterine rupture, postpartum hemorrhage, and urinary tract injury, posing serious threats to maternal and fetal safety [2,3,4]. Current clinical management of invasive PAS remains limited, primarily focusing on early diagnosis, close monitoring of disease progression, planned cesarean delivery at appropriate gestational ages, surgical removal of the implanted placenta and affected uterine tissues, and preoperative prophylactic placement of aortic balloons to reduce intraoperative bleeding [1,5,6,7]. However, effective interventions to halt the progression of PAS remain scarce, and the underlying pathogenic mechanisms are still not fully elucidated [8].

In clinical practice, the identification of biomarkers closely associated with PAS progression is of critical importance. Elucidating the pathogenesis of PAS may provide valuable insights into the mechanisms driving disease advancement. Current evidence suggests that the pathogenesis of PAS involves impaired decidualization, enhanced invasiveness of trophoblast cells, and aberrant remodeling of uterine spiral arteries [9]. In vitro studies have demonstrated that the invasiveness of trophoblast cells is significantly elevated in damaged decidua compared to intact decidua, whereas no significant difference is observed between repaired and normal decidua, indicating that decidual damage markedly promotes trophoblast invasion [10,11]. Trophoblast invasion into the uterine wall is essential for normal placental development; however, in PAS, extravillous trophoblasts (EVTs) exhibit enhanced invasiveness, leading to excessive penetration into the myometrium, mucosal layers, and even beyond [9,12]. This process resembles a cancer-like progression [9,12]. Such excessive invasion hinders normal placental separation during delivery, and manual removal may lead to life-threatening hemorrhage. Therefore, investigating subpopulations of trophoblasts with heightened invasive potential is essential for understanding the pathogenesis of PAS.

High-throughput technologies are instrumental in the discovery of disease biomarkers, facilitating early diagnosis, prognostic evaluation, and disease monitoring [13,14,15]. As a powerful high-throughput tool, mass spectrometry (MS)-based proteomics enable comprehensive identification and quantification of proteins, thereby providing an unbiased methodology for biomarker discovery and mechanistic insight [16,17,18]. In this study, we employed a data-independent acquisition (DIA)-based quantitative proteomic strategy to investigate whether women with PAS exhibit a distinct protein signature in maternal plasma.

In the subsequent analysis, we examined longitudinal blood samples from five PAS patients, obtained at 30 weeks of gestation and immediately prior to delivery (10 samples in total). The central objectives were to discover biomarkers strongly linked to PAS disease progression and to define the functional roles and mechanisms of these biomarkers in the progression of PAS.

## 2. Material and Methods

### 2.1. Clinical Sample Collection and Blood Processing

This study retrospectively enrolled patients with PAS from the Department of Obstetrics, Peking University First Hospital, between 2018 and 2020, as previously reported [19,20]. PAS was confirmed by trained reviewers through comprehensive chart review using established criteria: (a) Pathological diagnosis [21]: Microscopic evidence of chorionic villi attached to or invading the myometrium without an intervening decidual layer. (b) Clinical diagnosis [22]: Based on the FIGO classification. FIGO grade 1 featured abnormal placental attachment without uterine serosal invasion or neovascularization. FIGO grade 2 showed significant serosal hypervascularity without full-thickness invasion. FIGO grade 3 was defined by placental invasion beyond the uterine serosa into parametrial or adjacent organs. Patients meeting either criterion were included.

For immunohistochemistry analysis, a self-controlled design was adopted to minimize potential confounding factors such as different gestational ages and maternal comorbidities. Specifically, normal placental regions (villi and basal plate with intact structure taken from normally detached placenta) from the same PAS patient served as controls, while deeply implanted regions (where villi were deeply implanted into the uterine wall) were designated as the PAS group. This design effectively controls for inter-individual variability, allowing a more accurate assessment of KRT6A expression specifically associated with PAS pathology.

All data were extracted from inpatient medical records and encompassed the following categories: (1) demographic characteristics: maternal age; (2) obstetric history: gravidity, parity, and history of abortion, prior cesarean section; (3) current pregnancy characteristics: gestational age at delivery, presence of placenta previa, relevant comorbidities including gestational diabetes mellitus, hypertensive disorders of pregnancy, and thyroid dysfunction (hyperthyroidism or hypothyroidism); (4) ultrasound score: the ultrasound scoring system for PAS developed by Peking University First Hospital was adopted [23], and scoring was performed by two experienced sonographers.

Blood samples were obtained from preoperative tests conducted within the two weeks immediately preceding delivery. Peripheral blood was collected using 10 mL acid citrate dextrose (ACD) vacutainer tubes and centrifuged at 1500× *g* for 10 min. The resulting plasma was then carefully aliquoted into new centrifuge tubes and stored immediately at −80 °C [24].

### 2.2. Ethics Statement

The study was conducted in accordance with the Declaration of Helsinki. Ethical approval was obtained from the Institutional Review Ethics Committee of Peking University First Hospital (Approval No. 2020[411]).

### 2.3. Mass Spectrometry Measurements

The experimental workflow comprised multiple stages, including sample preparation for data-independent acquisition (DIA) analysis, sample processing for spectral library construction, and high-pH reversed-phase fractionation. Liquid chromatography was performed using a nanoElute system (Bruker Daltonics GmbH & Co. KG, Bremen, Germany). All samples were subjected to analysis on a hybrid trapped ion mobility spectrometry quadrupole time-of-flight mass spectrometer (TIMS-TOF Pro, Bruker Daltonics, Bremen, Germany) equipped with a CaptiveSpray nanoelectrospray ion source. Raw data were processed with a developmental version of Spectronaut (v14.0.200409.43655, Biognosys, Schlieren, Switzerland) [17].

### 2.4. Immunohistochemistry Pathological Examination of Placental Tissues

Serial sections of paraffin-embedded human placenta (fixed in 4% PFA) underwent antigen retrieval in citrate buffer (10 mM, pH 6.0) using a pressure cooker. After blocking with 10% normal goat serum (ZSGB, Beijing, China), sections were incubated overnight at 4 °C with an anti-KRT6A antibody (1:200, Abcam, Cambridge, UK, ab238013). Following peroxidase blockade, a secondary antibody (ZSGB, Beijing, China) was applied, and signals were developed with DAB prior to hematoxylin counterstaining. For quantitative analysis, three random fields per section were captured at 200× magnification. The mean optical density (MOD) was measured using ImageJ software (version 1.53t, National Institutes of Health, Bethesda, MD, USA). Specifically, the integrated optical density (IOD) and the positive stained area were measured, and the average optical density was calculated as IOD/Area. The average MOD of the three fields was calculated for each tissue section.

### 2.5. Multiplex Immunofluorescence Staining

Multiplex immunofluorescence staining was performed on formalin-fixed, paraffin-embedded placental tissue sections. Consecutive staining was carried out using heat-induced antigen retrieval followed by incubation with primary antibodies. The signals were amplified and detected with Opal™ polymer horseradish peroxidase and Opal fluorophores (Akoya Biosciences, Marlborough, MA, USA). Between each round of staining, sections were subjected to heat-induced antibody stripping using a microwave. The following primary antibodies and Opal channels were used in sequential order: Anti-KRT6A (1:100, Abcam, Cambridge, UK, ab238013 detected with Opal 520); Anti-VIM (1:200, Abcam, Cambridge, UK, ab8069 detected with Opal 620); Anti-KRT7 (1:200, Abcam, Cambridge, UK, ab68459 detected with Opal 570). All Opal reagents were used at a dilution of 1:100. Nuclei were counterstained with spectral DAPI. Slides were finally mounted in antifade mounting medium (Akoya Biosciences, Marlborough, MA, USA) and scanned using a Vectra Polaris system (Akoya Biosciences, Marlborough, MA, USA).

### 2.6. Cell Lines and Cell Culture

Human choriocarcinoma BeWo cells and HTR-8/SVneo cells were purchased from the National Infrastructure of Cell Line Resource (NICR) [25]. All cells were stored in liquid nitrogen and cultured in Roswell Park Memorial Institute (RPMI) 1640 medium (Sigma, St. Louis, MO, USA) supplemented with 1% antibiotic-antimycotic (Gibco, Grand Island, NY, USA) and 10% heat-inactivated fetal bovine serum (FBS, Gibco, Grand Island, NY, USA) in a humidified incubator (5% CO_2_ at 37 °C). These cell lines were authenticated with STR profiling and were also checked if they were free of mycoplasma contamination by polymerase chain reaction (PCR) and culture.

### 2.7. Establishment of KRT6A-Overexpressing Stable Cell Lines

The full-length human KRT6A coding sequence was synthesized and cloned into a lentiviral expression vector (GV492-Puro). Lentiviral particles were produced by co-transfecting HEK293T cells with the KRT6A expression plasmid and packaging plasmids (psPAX2 and pMD2.G) using Lipofectamine 3000 (Invitrogen, Waltham, MA, USA). The supernatant containing lentiviral particles was collected 48 h post-transfection, filtered through a 0.45 μm filter, and used to infect HTR-8/SVneo and BeWo cells. After 48 h of infection, stable transductants were selected with puromycin (1–2 μg/mL) for 7–14 days. The overexpression efficiency was validated by fluorescence microscopy, RT-qPCR, and Western blot.

### 2.8. Western Blot Analysis

Cells were lysed in RIPA buffer (Beyotime Biotechnology, Shanghai, China) supplemented with a protease-inhibitor cocktail (Roche, Basel, Switzerland). Protein concentrations were determined using a BCA protein assay kit (Thermo Fisher Scientific, Waltham, MA, USA). Equal amounts of protein (20–30 μg per lane) were separated by 10% SDS-PAGE and transferred onto PVDF membranes (Millipore, Burlington, MA, USA). The membranes were blocked with 5% non-fat milk in TBST (Tris-buffered saline with 0.1% Tween-20) for 1 h at room temperature, followed by overnight incubation at 4 °C with primary antibodies: anti-Flag (1:10,000, Sigma-Aldrich, St. Louis, MO, USA, F7425) and anti-GAPDH (1:2000, Abcam, Cambridge, UK, ab8245) as a loading control. After washing with TBST, the membranes were incubated with HRP-conjugated secondary antibodies (1:5000, ZSGB, Beijing, China) for 1 h at room temperature. Protein bands were visualized using an enhanced chemiluminescence (ECL) substrate (Millipore, Burlington, MA, USA) and imaged using a ChemiDoc system (Bio-Rad, Hercules, CA, USA).

### 2.9. RNA Extraction and Quantitative Real-Time PCR (RT-qPCR)

Total RNA was extracted from cultured cells using TRIzol reagent (Invitrogen, Waltham, MA, USA) according to the manufacturer’s instructions. RNA concentration and purity were assessed using a NanoDrop spectrophotometer (Thermo Fisher Scientific, Waltham, MA, USA). Reverse transcription was performed using a PrimeScript RT reagent kit (Takara Bio Inc., Kusatsu, Shiga, Japan) to synthesize complementary DNA (cDNA). Quantitative real-time PCR was carried out using SYBR Green Master Mix (Takara, Japan) on a LightCycler 480 system (Roche, Basel, Switzerland). The thermal cycling conditions were: 95 °C for 30 s, followed by 40 cycles of 95 °C for 5 s and 60 °C for 30 s. The relative expression levels of target genes were calculated using the 2^−ΔΔCt^ method, with GAPDH as an internal control. The primer sequences used in this study were as follows: GAPDH forward: 5′-GGAGCGAGATCCCTCCCAAAAT-3′, reverse: 5′-GGCTGTTGTTCATACTTCTCATTGG-3′; KRT6A forward: 5′-AATCGAATCCCACCATCCAGC-3′, reverse: 5′-CTCCAGGTTCTGCCCTCACAG-3′.

### 2.10. Flow Cytometry Assay

For the cell cycle analysis, harvested cells were fixed with 75% ice-cold ethanol in phosphate-buffered saline (PBS). Subsequently, the cells were treated with Bovine Pancreatic RNase (2 mg/mL; Sigma-Aldrich, St. Louis, MO, USA) and propidium iodide (10 mg/mL; Invitrogen, Waltham, MA, USA), followed by a 30 min incubation at room temperature in the dark. Cell cycle distribution was then analyzed using a flow cytometer (BD Biosciences, Franklin Lakes, NJ, USA). For the apoptosis assay, the experimental procedures were performed in accordance with the manufacturer’s protocol. Apoptotic cells were quantified by flow cytometry (BD Biosciences, Franklin Lakes, NJ, USA). In the resulting dot plot, the four quadrants represent necrotic cells, viable cells, early apoptotic cells, and late apoptotic cells, respectively.

### 2.11. Transwell Assay

Cells were seeded into the upper chamber at a density of 2 × 10^6^ cells per well in serum-free medium. The lower chamber was filled with 500 µL of culture medium containing 20% FBS. After incubation for 48 h at room temperature in a 5% CO_2_ atmosphere, non-invasive cells and Matrigel in the upper chamber were carefully removed. Cells that had migrated to the lower surface were fixed with 10% neutral buffered formalin and stained with 0.1% crystal violet. The number of invading cells was counted in five randomly selected fields under a microscope.

### 2.12. Migration and Invasion Assays (Scratch-Based)

Cells in the logarithmic growth phase (passages 3–5) were seeded into six-well plates at a density of 1 × 10^6^ cells per milliliter and incubated for 24 h. When the cells reached approximately 70% confluency, a uniform scratch was created across the monolayer using a sterile 10 μL pipette tip. After scratching, the cells were gently washed twice with PBS to remove any detached cells.

For the migration assay, the plates were then placed in an IncuCyte live-cell imaging system and cultured under standard conditions (37 °C, 5% CO_2_). Wound closure was monitored and imaged at 0, 24, 48, and 72 h. For the invasion assay, after creating the scratch wound and washing with PBS, a thin layer of Matrigel (Corning, NY, USA) was applied to the wells. The Matrigel layer serves as an extracellular matrix barrier, and only cells capable of degrading and invading through the Matrigel can close the wound. The plates were then placed in the IncuCyte system, and wound closure was monitored and imaged at the same time points (0, 24, 48, and 72 h).

### 2.13. Bioinformatics Analysis

The proteomic data were imputed based on the protein-detecting rate determined in previous research [17]. The imputed data were then normalized using the LogNorm algorithm. Principal component analysis (PCA) was employed to perform clustering analysis of the samples. The fold-change values of proteins were calculated using the R package Genefilter. Differentially expressed proteins were filtered based on a *p* value (*t* test) < 0.05 and |log(fold change)| > 0.6. Compound and enzyme relationships were obtained from the Kyoto Encyclopedia of Genes and Genomes (KEGG) ligand database. Venn diagrams, heatmaps, and network visualizations were created using the ggplot2 packages [26]. The z score algorithm was applied to predict the activation state (either activated or inhibited) of biological processes. Single-cell RNA sequencing (scRNA-Seq) data of PAS were downloaded from the Genome Sequence Archive (Project No. PRJCA008155) [27]. The Seurat package [28] was used for annotating cell types. The Cancer Genome Atlas (TCGA) and the Genotype-Tissue Expression (GTEx) databases were utilized to analyze pan-cancer KRT6A expression profiles and their associated signaling pathways.

### 2.14. Statistical Analysis

The normality of data distribution was assessed using appropriate tests (e.g., Shapiro–Wilk test). For data following a normal distribution, results were presented as mean ± standard deviation (SD), and comparisons between two groups were performed using independent samples *t*-test. For data not following a normal distribution, results were presented as median with interquartile range (IQR), and comparisons between two groups were performed using the Mann–Whitney U test. Categorical data were expressed as counts and percentages, and group comparisons were conducted using the χ^2^ test or Fisher’s exact test, as appropriate. A *p*-value < 0.05 was considered statistically significant. All data were analyzed, plotted, or visualized using R software (version 4.1.0) or the ggplot2 package [26].

## 3. Results

### 3.1. Demographic Characteristics of the Study Population

**Figure 1:**
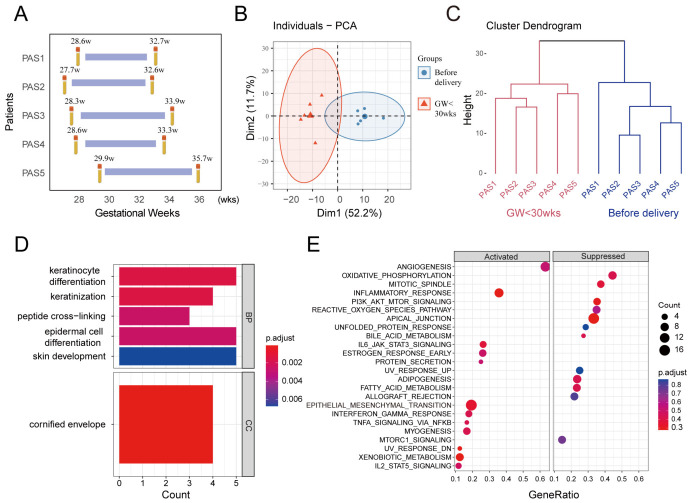
Proteomic profiling of placenta accreta spectrum (PAS) across different gestational ages. (**A**) Timeline flowchart illustrating the five sampling sessions for PAS specimens. Principal component analysis (**B**) and hierarchical clustering (**C**) revealed distinct inter-group variation following data normalization, demonstrating clear stratification and effective discrimination between the two groups. Functional enrichment analysis, including Gene Ontology (**D**) and Gene Set Enrichment Analysis (**E**), was performed to compare the two PAS groups (pre-delivery and gestational age < 30 weeks).

To explore maternal plasma biomarkers associated with the progression of PAS, this study incorporated a total of 10 blood samples for DIA proteomic analysis, and the raw data are available in the supplementary table. The samples were obtained from five PAS cases at different gestational ages, with each patient providing one sample before 30 weeks of gestation and another prior to delivery. All five PAS cases had a history of prior cesarean delivery (100%) (Table 1). The specific gestational ages at sampling were as follows: Case 1, 28.6–32.7 weeks; Case 2, 27.7–32.6 weeks; Case 3, 28.3–33.9 weeks; Case 4, 28.6–33.3 weeks; Case 5, 29.9–35.7 weeks (Figure 1A). Disease progression was observed in all cases with advancing gestation, as evidenced by higher ultrasound scores before delivery compared to those before 30 weeks. Specifically, the ultrasound scores increased from 10 to 13 in PAS1, from 9 to 12 in PAS2, from 11 to 14 in PAS3, from 9 to 12 in PAS4, and from 11 to 15 in PAS5. Additionally, clinical information was collected from another cohort (N = 14) used for histological validation of PAS (Supplementary Table S1).

### 3.2. Proteins in PAS Plasma with Significantly Altered Expression Levels Associated with Disease Progression

Our data revealed distinct stratification among the groups based on principal component analysis (Figure 1B) and hierarchical clustering (Figure 1C). In comparison to samples collected before 30 weeks of gestation, the progression of PAS was associated with the identification of 26 differentially expressed proteins in maternal plasma prior to delivery (Table 2). Among these, 46.2% (12/26) were significantly up-regulated (*p* < 0.05), whereas the remaining 53.8% (14/26) exhibited significant down-regulation (*p* < 0.05).

To explore the dysregulated biological processes occurring with the progression of PAS, this study conducted GO Analysis on the differential proteins. The results showed that as PAS progresses, compared to the 30th week of gestation, abnormal biological processes in PAS before delivery include cell keratinization, keratinocyte differentiation, epidermal cell differentiation, and cornification of keratinocytes (Figure 1D). The GSEA analysis showed that compared to before 30 weeks of gestation, biological processes abnormally activated in PAS before delivery included angiogenesis, epithelial–mesenchymal transition, myogenesis, while those abnormally inhibited included oxidative phosphorylation, PI3K_AKT_MTOR signaling pathway, etc. (Figure 1E).

### 3.3. The KRT6A, Specifically Expressed in Trophoblast Cells, Showed Increased Expression with the Progression of PAS Disease

**Figure 2:**
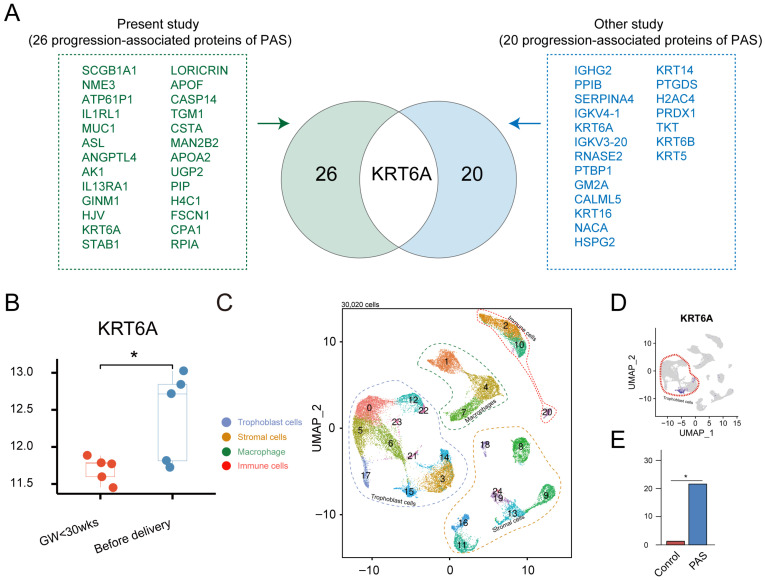
Cross-validation of KRT6A as a hub protein in placenta accreta spectrum (PAS). (**A**) Venn diagram showing the intersection between two independent proteomic datasets. The left circle represents the 26 differentially expressed proteins identified in the present study by comparing pre-30-week and pre-delivery plasma samples from the same five PAS patients (longitudinal progression-associated proteins). The right circle represents the 20 differentially expressed proteins reported in a previous study [29] that compared term PAS patients with healthy controls (cross-sectional PAS-associated proteins). KRT6A is the only overlapping protein between the two datasets. (**B**) Assessment of KRT6A expression across PAS groups (pre-delivery vs. gestational age < 30 weeks). (**C**) UMAP plot showing the unbiased cells clusters in the maternal–fetal interface using an independent published dataset. (**D**) KRT6A is specifically highly expressed in trophoblasts at the maternal–fetal interface in PAS. (**E**) Quantitative analysis of KRT6A expression in the single-cell dataset, comparing deeply implanted regions (PAS) versus normal placental regions (control) from the same PAS patients (self-controlled design). KRT6A expression was significantly higher in the PAS group (* *p* < 0.05).

Subsequently, the panel of 26 differentially expressed proteins (identified by comparing pre-30-week and pre-delivery samples from the same PAS patients) was further refined. To distinguish proteins specifically associated with PAS progression from those that merely reflect normal gestational changes, we performed an intersection analysis with a previously published proteomic dataset that compared 15 PAS patients with 15 healthy controls at delivery using plasma proteomic analysis (LC-MS/MS), identifying 20 differentially expressed proteins associated with the presence of PAS [29]. As shown in the Venn diagram (Figure 2A), the overlapping region between the two datasets contained only one protein: KRT6A. As shown in Figure 2B, the statistical summary of KRT6A levels in the five PAS patients revealed a general upward trend from the pre-30-week time point to before delivery. However, individual patient trajectories showed some heterogeneity: three patients exhibited a pronounced increase in KRT6A levels, while the remaining two showed relatively stable expression. Despite this inter-individual variation, the overall trend across the cohort reached statistical significance (* *p* < 0.05), indicating that KRT6A upregulation is associated with PAS progression at the population level.

To investigate cell type-specific expression patterns, we analyzed an independent published single-cell RNA-sequencing dataset that profiled the cellular landscape of PAS placenta using a self-controlled design [27]. In that study, normal placental regions from the same PAS patient served as controls, while deeply implanted regions were designated as the PAS group [27]. As shown in the UMAP plot (Figure 2C), KRT6A was localized to trophoblast clusters at the maternal–fetal interface in PAS (Figure 2D). Moreover, within trophoblast populations, KRT6A expression levels were elevated in PAS samples relative to controls (Figure 2E).

### 3.4. KRT6A Participates in Enhancing the Migration and Invasive Capability of Trophoblast Cells

**Figure 3:**
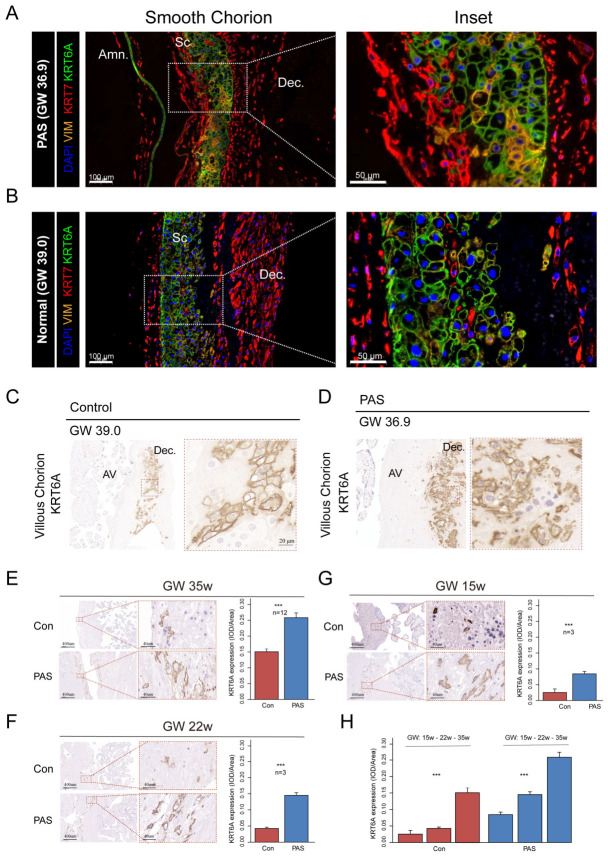
Immunohistochemical detection of KRT6A-positive trophoblasts in the maternal–fetal interface. (**A**,**B**) Representative images of smooth chorion showing KRT6A-positive trophoblasts localized in the chorion between the amnion and decidua. Scale bar = 50 μm and 100 μm. (**C**,**D**) Representative images of villous chorion showing KRT6A-positive cells predominantly localized in the decidua. Scale bar = 20 μm and 200 μm. (**E**–**G**) Quantitative analysis of KRT6A expression levels in PAS and control tissues at different gestational stages: 35 weeks (**E**), 22 weeks (**F**), and 15 weeks (**G**). Scale bar = 50 μm and 100 μm. (**H**) Longitudinal analysis within the PAS and control group showing a progressive increase in KRT6A expression with advancing gestation (15, 22, and 35 weeks). Amn, amnion; Dec, decidua; SC, smooth chorion; VC, villous chorion. (*** *p* < 0.001).

To further investigate the localization and quantitative expression of KRT6A, immunohistochemistry and immunofluorescence staining were performed at the maternal–fetal interface. As shown in Figure 3A,B, in the smooth chorion, KRT6A-positive trophoblasts were localized within the chorion between the amnion and decidua. In the villous chorion (Figure 3C,D), KRT6A-positive cells were predominantly localized within the decidua. Quantitative analysis of KRT6A immunostaining revealed significantly higher expression levels in PAS tissues compared to controls across different gestational stages, including late gestation at 35 weeks (Figure 3E), mid-gestation at 22 weeks (Figure 3F), and mid-gestation at 15 weeks (Figure 3G). Furthermore, longitudinal analysis within the PAS group showed a progressive increase in KRT6A expression with advancing gestation (Figure 3H). The detailed information of the PAS specimens is provided in Supplementary Table S1.

**Figure 4:**
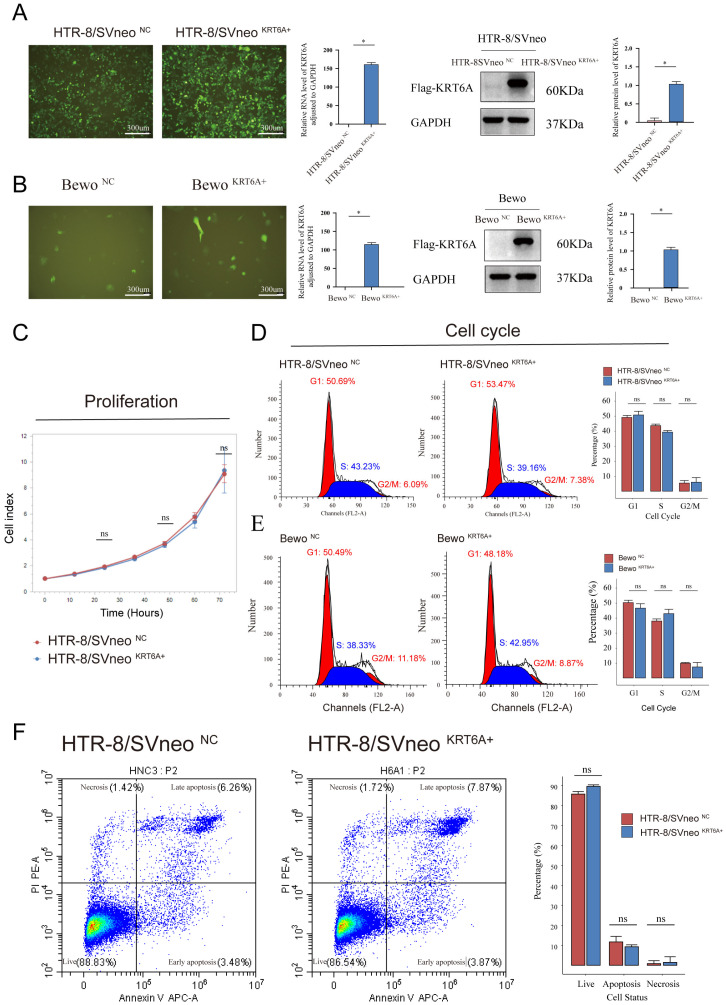
Effects of KRT6A overexpression on trophoblast proliferation, cell cycle, and apoptosis in vitro. (**A**,**B**) Efficiency of KRT6A overexpression in HTR-8/SVneo and Bewo cells, as confirmed by fluorescence microscopy, RT-qPCR, and Western blot. (**C**) Real-time proliferation kinetics monitored by the IncuCyte system. (**D**,**E**) Cell cycle distribution analyzed by flow cytometry in HTR-8/SVneo and Bewo cells following KRT6A overexpression. (**F**) Apoptosis rate in HTR-8/SVneo cells measured by flow cytometry. * *p* < 0.05; ns, not significant.

To further explore the role of KRT6A in the development of PAS, this study conducted in vitro experiments. HTR-8/SVneo and Bewo trophoblast cells were infected using lentivirus-mediated methods and stable cell lines overexpressing KRT6A were obtained through antibiotic selection. Results from fluorescent microscopy, RT-qPCR and Western blot analysis (Figure 4A,B) all demonstrated the successful establishment of stable KRT6A-overexpressing HTR-8/SVneo and Bewo cell lines. Next, the impact of KRT6A overexpression on trophoblast proliferation was further examined. The results showed that overexpression of KRT6A did not affect the proliferation capacity of HTR-8/SVneo cells (Figure 4C). Moreover, KRT6A overexpression did not significantly affect the cell cycle of HTR-8/SVneo cells (Figure 4D) or Bewo cells (Figure 4E). Next, flow cytometry was used to detect the proportion of apoptotic cells. The results showed that overexpression of KRT6A did not affect the proportion of apoptotic cells in HTR-8/SVneo cells (Figure 4F).

**Figure 5:**
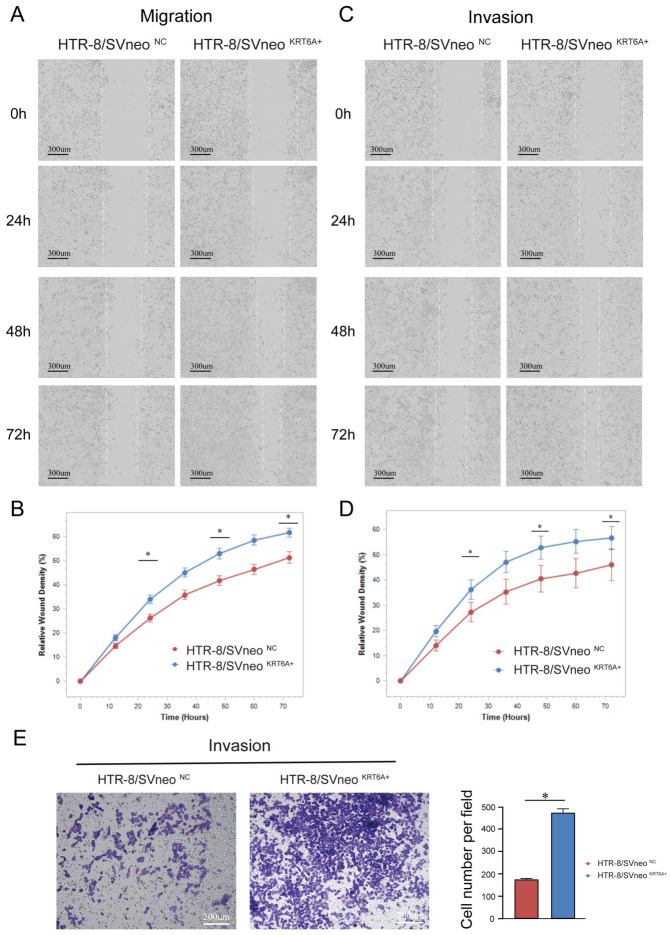
The effect of KRT6A overexpression on the migration and invasion capability of HTR-8/SVneo trophoblast cells. (**A**,**B**) Scratch migration assay (without Matrigel) and its quantification at 0, 24, 48, and 72 h (IncuCyte). Dashed lines indicate the leading edges of migrating or invading cells at the wound boundary. (**C**,**D**) Planar invasion assay (scratch wound with Matrigel coating) and its quantification at 0, 24, 48, and 72 h (IncuCyte). (**E**) Transwell invasion assay and its quantification. * *p* < 0.05. Scale bar = 300 μm.

Next, scratch assay results showed that KRT6A overexpression significantly enhanced the migration ability of HTR-8/SVneo cells (*p* < 0.05) (Figure 5A). Meanwhile, the invasion capability of HTR-8/SVneo trophoblast cells (Figure 5B,C) and Bewo trophoblast cells (Figure 5D) was significantly enhanced after KRT6A overexpression (*p* < 0.05). These cellular experiments collectively indicate that KRT6A overexpression indeed significantly increases trophoblast cell migration and invasion capabilities.

### 3.5. KRT6A Is Associated with MYC Signaling Pathway Activation

**Figure 6:**
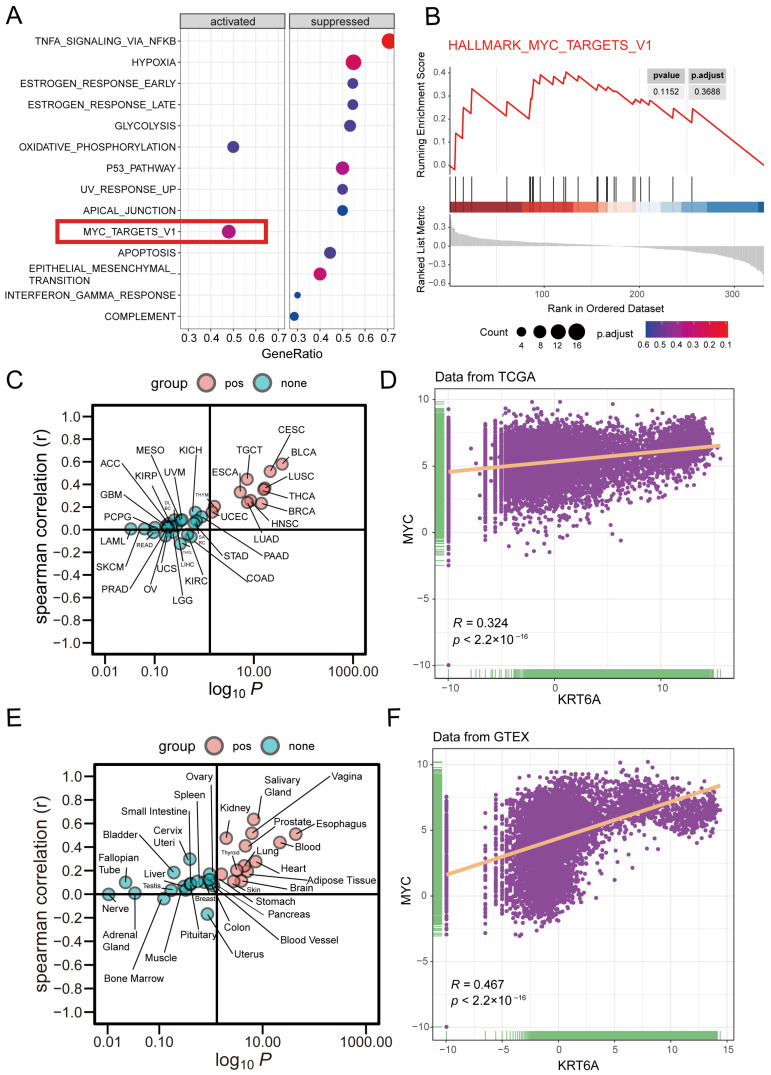
Expression patterns of KRT6A in pan-cancer analysis and its association with MYC signaling. (**A**) Gene Set Enrichment Analysis revealing signaling pathways altered during PAS progression. (**B**) Enhanced activation of the MYC pathway in KRT6A-positive trophoblasts from PAS samples compared to controls. (**C**,**D**) Correlation between KRT6A mRNA expression and MYC levels across 22 tumor types based on TCGA database. (**E**,**F**) Correlation between KRT6A mRNA expression and MYC levels across 22 tumor types based on GTEx database.

Furthermore, we have conducted a preliminary investigation into the mechanism by which KRT6A leads to an increase in the invasive ability of trophoblast cells using the previous study [27]. GSEA analysis showed that MYC_TAGRETS_V1 signal pathway was activated in the KRT6A^+^ trophoblast cells (Figure 6A,B). Given the resemblance between trophoblast cell implantation into the maternal uterus and tumor invasion, we performed a pan-cancer analysis of KRT6A expression and its correlation with MYC signaling using data from the TCGA and GTEx databases. In the TCGA database, KRT6A expression was strongly positively correlated with MYC across 22 cancer types (correlation coefficient: 0.324, *p* < 2.2 × 10^−16^) (Figure 6C,D). A similar positive correlation was observed in the GTEx database (correlation coefficient: 0.467, *p* < 2.2 × 10^−16^), further supporting the link between KRT6A and MYC signaling (Figure 6E,F).

## 4. Discussion

In humans, the differentiation from cytotrophoblasts (CTB) to extravillous trophoblasts (EVTs) is crucial for successful pregnancy. EVTs invade into the uterine decidua, remodel spiral arteries, and facilitate maternal blood flow into the intervillous space successfully [30]. In PAS, the increased invasive capacity of EVT leads to excessive invasion of the myometrium, and in severe cases, even penetration through the serosa and invasion into the bladder. This excessive invasion results in the placenta being unable to detach naturally during delivery, and if manual removal is required, it may lead to uncontrollable hemorrhage [9,12]. Currently, effective interventions to intervene in the progression of PAS are lacking. Therefore, exploring the key factor is crucial for exploring targets for intervening in the progression of PAS. The article explores molecule closely associated with the progression of PAS and investigates its function, may provide a potential target for intervening in the progression of PAS.

In the present study, using an unbiased plasma proteomic approach combined with functional assays, we identified KRT6A as a circulating biomarker that enhances trophoblast cell invasion and correlates with PAS progression. Of note, our previous single-cell RNA-sequencing study on PAS placenta independently identified KRT6A as a marker enriched in a subpopulation of cytotrophoblasts transitioning to extravillous trophoblasts along the differentiation trajectory [31]. That study revealed the cellular landscape and differentiation hierarchy of PAS at the transcriptomic level but did not examine the plasma protein levels or the direct functional role of KRT6A in promoting invasion. Taken together, these two complementary studies—one at the single-cell transcriptomic level and the other at the plasma proteomic and functional level—convergently support KRT6A as both a differentiation-associated marker and a potential functional driver of PAS progression. Thus, KRT6A may provide a potential target for intervening in the progression of PAS. Previous study [29] identified multiple keratins (e.g., KRT16, KRT5, KRT6B, etc.) as differentially expressed between PAS patients and healthy controls. However, in our longitudinal cohort, only KRT6A exhibited a progressive increase from pre-30-week to pre-delivery. The other keratins, although associated with the presence of PAS, did not show dynamic changes with disease progression. This explains why KRT6A was the sole overlapping protein. Consistently, our GO analysis showed that keratin-related biological processes were enriched during PAS progression (Figure 1D), supporting the relevance of this protein family. Nevertheless, among these keratins, only KRT6A was functionally validated to directly enhance trophoblast cell invasion (Figure 5), highlighting its specific role in driving PAS progression rather than merely serving as a passive marker.

KRT6A is a type II keratin involved in the epidermal keratinization process [32,33] and plays a crucial role in epithelial cell migration and tissue integrity maintenance [34]. Studies have shown that KRT6A participates in cell migration, particularly in the migration of keratinocytes involved in cornification [35]. In cancer research, silencing KRT6A leads to decreased expression of MMP2 and MMP9, while the expression of tissue inhibitor of metalloproteinases 2 (TIMP2) increases, thereby inhibiting the invasion and metastasis of nasopharyngeal carcinoma cells. This process mainly relies on the activation of the Wnt/β-catenin pathway to induce the epithelial–mesenchymal transition (EMT) process [36]. In lung cancer, KRT6A can also promote the growth and metastasis of lung adenocarcinoma by inducing the EMT process [37,38]. Additionally, KRT6A can promote the growth and invasion of lung cancer cells by activating the MYC signaling pathway [38]. Moreover, high expression of KRT6A is closely associated with poor prognosis in lung cancer patients [39,40]. This suggests that apart from PAS, KRT6A is also a key regulator of cell invasion and migration in other contexts. Elevated expression of KRT6A enhances the invasion ability of trophoblasts, which may be an important factor contributing to the increased peripheral blood KRT6A expression as PAS progresses and placental invasion deepens.

Given the well-established biological parallels between trophoblast invasion and tumor cell invasion, and the lack of large-scale public multi-omics databases for PAS, we sought to test the generalizability of the KRT6A-MYC association using the TCGA and GTEx pan-cancer datasets. The positive correlation identified across over 20 cancer types from two independent databases provides supportive evidence that the KRT6A-MYC association is not a spurious finding limited to our small PAS cohort, but rather a robust biological relationship. This analysis was intended as an exploratory validation of the generalizability of our findings, not as primary evidence.

A notable strength of this study is the use of a self-controlled design for immunohistochemical validation. In PAS patients, the placenta displays a continuous spectrum from normal attachment to deeply invasive regions. This unique landscape allows collection of both diseased and control tissues from the same individual. PAS patients often deliver preterm, making gestational age-matched healthy controls difficult to obtain, while alternative controls (e.g., preterm births or placenta previa without accreta) may introduce additional confounding. By using autologous normal tissue from the same patient as the control, this design minimizes common confounding factors in case–control studies, such as differences in gestational age, genetic background, and maternal comorbidities. Thus, it provides a rigorous assessment of molecular changes specifically associated with PAS progression and reduces the risk of spurious associations driven by inter-individual heterogeneity.

Currently, the diagnosis and treatment principles of PAS involve close monitoring of disease progression. This study provides a peripheral blood biomarker, KRT6A, for monitoring disease progression. If imaging methods are insufficient for diagnosing PAS, peripheral blood biomarkers such as KRT6A can be used as supplementary diagnostic tools. Once abnormal elevation of KRT6A expression is detected, the possibility of PAS occurrence and progression should be considered. For invasive and rapidly progressing PAS cases, timely referral to medical centers with multidisciplinary teams and rich experience in PAS management, as well as termination of pregnancy at the optimal timing, can minimize adverse outcomes associated with PAS [1,5,6,7].

## 5. Limitations of the Study

This study has certain limitations. Firstly, due to the relatively low incidence rate of PAS (<1%), the number of enrolled PAS patients was limited, and the small sample size may restrict the generalizability of the conclusions drawn in this study. As shown in Figure 2B, inter-individual heterogeneity was observed: three of the five PAS patients exhibited a pronounced increase in KRT6A levels, whereas the remaining two showed relatively stable expression. This variation may be attributable to the limited sample size or to the multifactorial nature of PAS pathogenesis, in which different molecular drivers may dominate in different patients. In the future, larger independent cohorts are needed to validate the clinical utility of KRT6A as a biomarker for PAS progression and to better characterize the spectrum of KRT6A responses across different patient subgroups.

We acknowledge that the pan-cancer analysis using TCGA and GTEx databases does not directly validate the KRT6A-MYC pathway in PAS tissues. Given the absence of large-scale public PAS datasets, this approach provided the best available means to test the generalizability of our findings. However, future studies using PAS-specific samples, including placental tissues and trophoblast cell lines, are needed to further confirm that the MYC signaling pathway is indeed activated downstream of KRT6A in the context of PAS progression.

Additionally, since many of the PAS patients in our hospital were transferred from other hospitals for delivery before childbirth, there were no blood samples available for the entire pregnancy (including early, mid, and late stages of pregnancy). Therefore, this study only included blood samples collected at 30 weeks before delivery and just before delivery from the existing specimen repository. In the future, further investigation will be conducted into the longitudinal changes of biomarkers throughout the entire pregnancy of PAS patients, aiming to diagnose and grade PAS at earlier stages. Serial measurements of KRT6A combined with ultrasound findings could also be explored as a dynamic monitoring strategy for disease progression. Furthermore, there will be further exploration of key proteins that change with the progression of PAS, and the changes in cellular heterogeneity at the maternal–fetal interface of PAS will be verified through more in vitro and in vivo experiments, along with their reflection in peripheral blood biomarkers.

## 6. Conclusions

In summary, plasma biomarkers hold promise for future application in prenatal grading and prediction of adverse outcomes in PAS. They can aid in the refinement of perioperative management of PAS and facilitate the development of potential interventions targeting key processes in its development. This could shift the diagnostic and therapeutic paradigm of PAS from “early diagnosis, early grading” to “predictable, intervenable,” thereby providing tangible benefits to PAS patients.

## Figures and Tables

**Table 1:** Clinical information of the 5 PAS patients from the proteomics analysis.

Patients	PAS1	PAS2	PAS3	PAS4	PAS5
Maternal age (year)	33	37	30	34	31
Gravidity	1	2	1	3	3
Parity	1	1	1	1	0
Previous Cesarean section history	Yes	Yes	Yes	Yes	Yes
Placenta previa	Yes	Yes	Yes	Yes	Yes
Gestational diabetes mellitus	-	-	-	-	-
Gestational hypertension disorder	-	-	-	-	-
Twin pregnancy	-	-	-	-	-
Sampling gestational age (before 30 weeks, weeks)	28.6	27.7	28.3	28.6	29.9
Sampling gestational age (before delivery, weeks)	32.7	32.6	33.9	33.3	35.7
Ultrasound score (before 30 weeks)	10	9	11	9	11
Ultrasound score (before delivery, weeks)	13	12	14	12	15

**Table 2:** Expression profiles of 26 differential proteins.

Protein Name	Full Protein Name	logFC	Changes in PAS	*p* Value
SCGB1A1	Secretoglobin Family 1A Member 1	7.250	Up	0.004
NME3	Nucleoside Diphosphate Kinase 3	7.160	Up	0.008
ATP6AP1	Atpase H+ Transporting Accessory Protein 1	6.324	Up	0.002
IL1RL1	Interleukin 1 Receptor-Like 1	6.256	Up	0.034
MUC1	Mucin 1	5.764	Up	0.033
ASL	Argininosuccinate Lyase	5.283	Up	0.033
ANGPTL4	Angiopoietin-Like 4	5.253	Up	0.033
AK1	Adenylate Kinase 1	5.110	Up	0.033
IL13RA1	Interleukin-13 Receptor Subunit Alpha 1	5.055	Up	0.033
GINM1	Glycoprotein Integral Membrane Protein 1	4.964	Up	0.033
HJV	Hemojuvelin	4.626	Up	0.033
KRT6A	Keratin 6A	0.724	Up	0.029
STAB1	Stabilin-1	−0.402	Down	0.038
LORICRIN	Loricrin	−0.421	Down	0.049
APOF	Apolipoprotein F	−0.540	Down	0.043
CASP14	Caspase 14	−0.545	Down	0.018
TGM1	Transglutaminase 1	−0.601	Down	0.021
CSTA	Cystatin A	−0.704	Down	0.033
MAN2B2	Mannosidase Alpha Class 2b Member 2	−0.760	Down	0.021
APOA2	Apolipoprotein A-II	−0.940	Down	0.027
UGP2	UDP-Glucose Pyrophosphorylase 2	−1.211	Down	0.006
PIP	Peripheral Intrinsic Protein	−1.225	Down	0.049
H4C1	Histone Cluster 1	−1.405	Down	0.010
FSCN1	Fascin Actin-Bundling Protein 1	−4.975	Down	0.034
CPA1	Carboxypeptidase A1	−5.386	Down	0.033
RPIA	Ribose-5-Phosphate Isomerase A	−5.558	Down	0.034

## Data Availability

The mass spectrometry data are listed in Supplementary Table S2. The original contributions presented in this study are included in the article/Supplementary Material. Further inquiries can be directed to the corresponding author.

## Supplementary Materials

The following supporting information can be downloaded at: https://www.mdpi.com/article/10.3390/cells15131204/s1. Table S1: Clinical information of PAS patients for histochemical verification (N = 18); Table S2: Raw data of the proteomics analysis.
