# Supplementary material for: KRT6A Is a Biomarker of PAS Progression and Enhances the Invasive Ability of Trophoblast Cells
Source: Cells. 2026 Jul 2;15(13):1204. doi: 10.3390/cells15131204 (PMC13359832; doi:10.3390/cells15131204)
Supplement: Supplementary file 1 [file cells-15-01204-s001.zip › cells-4284109-Supplementary Tables/cells-4284109-Table S1.pdf]

Supplementary Table 1 Clinical information of PAS patients for histochemical verification (N=14)

	Second trimester		Third trimester
Cases	PAS8-10	PAS11-13	PAS14-25
Maternal age [median (IQR)], y	35 [33, 42]	35 [34, 37]	33 [32, 36]
Gestational weeks [median (IQR)], wk	15.2[15.0,15.4]	22.5 [22, 23]	34.0 [33.4, 34.5]
Pregnancy history			
Previous cesarean delivery/ (%)	3（100.0）	3（100.0）	12（100.0）
Gravidity (times)/ [median (IQR)]	3 [3, 4]	3 [3, 3]	3 [2, 3]
Parity (times)/ [median (IQR)]	1 [1, 1]	1 [1, 1]	1 [1, 1]
History of abortion/ (%)	2（100.0）	3（100.0）	11（91.7）
Pregnancy complications			
Placenta previa (Yes/no)/ (%)	-	-	12（100.0）
Hypothyroidism (Yes/no)/ (%)	0（0.0）	0（0.0）	0（0.0）
Hyperthyroidism (Yes/no)/ (%)	0（0.0）	0（0.0）	0（0.0）
Gestational diabetes mellitus/ (%)	0（0.0）	0（0.0）	0（0.0）
Hypertensive disorders of pregnancy/ (%)	0（0.0）	0（0.0）	0（0.0）
Fetal growth restriction/ (%)	0（0.0）	0（0.0）	0（0.0）
